# Automated interpretation of ANCA patterns - a new approach in the serology of ANCA-associated vasculitis

**DOI:** 10.1186/ar4119

**Published:** 2012-12-14

**Authors:** Ilka Knütter, Rico Hiemann, Therese Brumma, Thomas Büttner, Kai Großmann, Marco Cusini, Francesca Pregnolato, Maria Orietta Borghi, Ursula Anderer, Karsten Conrad, Dirk Reinhold, Dirk Roggenbuck, Elena Csernok

**Affiliations:** 1 Research and Development Department, GA Generic Assays GmbH, Ludwig-Erhard-Ring 3, 15827 Dahlewitz/Berlin, Germany; 2 Faculty of Science, Lausitz University of Applied Sciences, Großenhainer Str. 57, 01968 Senftenberg, Germany; 3 Department of Dermatology, Fondazione Ca Granda Ospedale Maggiore Policlinico, via S. Barnaba 8, 20122 Milan, Italy; 4 IRCCS Istituto Auxologico Italiano, Immune research laboratory and Department of Clinical Science and Community, University of Milan, via Spagnoletto 3, 20149 Milan, Italy; 5 Institute of Immunology, Technical University Dresden, Fiedler Str. 42, 01307 Dresden, Germany; 6 Institute of Molecular and Clinical Immunology, Otto-von-Guericke-University, Leipziger Str. 44, 39120 Magdeburg, Germany; 7 Department of Rheumatology, University of Schleswig-Holstein Campus Lübeck and Rheumaklinik Bad Bramstedt, Oskar-Alexander-Straße 26, 24576 Bad Bramstedt, Germany

## Abstract

**Introduction:**

Indirect immunofluorescence (IIF) employing ethanol-fixed neutrophils (ethN) is still the method of choice for assessing antineutrophil cytoplasmic antibodies (ANCA) in ANCA-associated vasculitides (AAV). However, conventional fluorescence microscopy is subjective and prone to high variability. The objective of this study was to evaluate novel pattern recognition algorithms for the standardized automated interpretation of ANCA patterns.

**Methods:**

Seventy ANCA-positive samples (20 antimyeloperoxidase ANCA, 50 antiproteinase3 ANCA) and 100 controls from healthy individuals analyzed on ethN and formalin-fixed neutrophils (formN) by IIF were used as a 'training set' for the development of pattern recognition algorithms. Sera from 342 patients ('test set') with AAV and other systemic rheumatic and infectious diseases were tested for ANCA patterns using the novel pattern recognition algorithms and conventional fluorescence microscopy.

**Results:**

Interpretation software employing pattern recognition algorithms was developed enabling positive/negative discrimination and classification of cytoplasmic ANCA (C-ANCA) and perinuclear ANCA (P-ANCA). Comparison of visual reading of the 'test set' samples with automated interpretation revealed Cohen's kappa (κ) values of 0.955 on ethN and 0.929 on formN for positive/negative discrimination. Analysis of the 'test set' with regard to the discrimination between C-ANCA and P-ANCA patterns showed a high agreement for ethN (κ = 0.746) and formN (κ = 0.847). There was no significant difference between visual and automated interpretation regarding positive/negative discrimination on ethN and formN, as well as ANCA pattern recognition (*P *> 0.05, respectively).

**Conclusions:**

Pattern recognition algorithms can assist in the automated interpretation of ANCA IIF. Automated reading of ethN and formN IIF patterns demonstrated high consistency with visual ANCA assessment.

## Introduction

Antineutrophil cytoplasmic antibodies (ANCA)-associated systemic small vessel vasculitis (AAV) comprising granulomatosis with polyangiitis (GPA, previously known as Wegener's granulomatosis, microscopic polyangiitis (MPA), and eosinophilic granulomatosis with polyangiitis (EGPA), previously known as Churg-Strauss syndrome, is a group of related autoimmune disorders characterized by microvascular inflammation, tissue necrosis, and circulating ANCA [1-6]. According to the recommendations for ANCA diagnostics, positive findings of standard screening tests by indirect immunofluorescence (IIF) on ethanol-fixed neutrophils (ethN) need to be confirmed with antigen-specific enzyme-linked immunosorbent assays (ELISAs) [4]. Dependent on ethN IIF pattern, ANCA can be subclassified into cytoplasmic ANCA (C-ANCA) and perinuclear ANCA (P-ANCA) patterns. Non-C/P-ANCA patterns are usually reported as atypical ANCA, which have been found in particular in patients with inflammatory bowel disease [7-9]. The majority of C-ANCA recognizes proteinase 3 (PR3) and a positive C-ANCA pattern confirmed by an anti-PR3-ANCA ELISA is pathognomonic for GPA [1,3]. In contrast, the main autoantigenic target of P-ANCA is myeloperoxidase (MPO) and such ANCA have been demonstrated in patients with MPA, EGPA and less frequently in Goodpasture's syndrome patients. Furthermore, the titer of both anti-PR3-ANCA and anti-MPO-ANCA is strongly associated with the active and inactive state of GPA and MPA, respectively. Due to the observations that anti-MPO-ANCA and antinuclear antibodies (ANAs) may demonstrate similar IIF patterns on ethN, IIF on formalin-fixed neutrophils (formN) is employed for their discrimination [10].

Pattern interpretation of ANCA is characterized by human bias and high variability due to methodological issues such as differing fixation protocols for neutrophils and fluorescence microscopy components (for example, lamps, filters, objectives) [11]. Remarkably, computer-based image analysis of IIF patterns by pattern recognition algorithms has recently been successfully applied for automated analysis of ANA by HEp-2 cell-based assays [12-14], of dsDNA antibodies by *Crithidia *cell-based assays and of ANCA by neutrophil cell-based assays [15,16]. However, the study of Melegari *et al. *[16] published as a review covered a small number of samples and only positive/negative discrimination between manual and automated ANCA pattern interpretation. Interestingly, Boomsma *et al. *reported earlier an IIF method for the quantitative image analysis of anti-PR3 antibody positive GPA patients [17]. The study did not reveal major differences between quantitative image analysis and the other techniques including ELISA and titration by manual IIF in their capacity to predict relapses of disease activity. However, no comprehensive approach using pattern recognition algorithms for automated ANCA pattern interpretation like in the present study has been reported so far. Furthermore, we provide for the first time variability data of an automated ANCA IIF pattern interpretation in the present study. In particular, a novel pattern recognition algorithm software module for ANCA pattern analysis has been established on the automated reading system AKLIDES™ and was compared to conventional routine interpretation of ANCA by IIF on ethN and formN.

## Materials and methods

### Patients

Seventy ANCA positive samples with distinct ANCA specificities (20 anti-MPO-ANCA, 7 males, 13 females, median age 68 years, range 57 to 74 years and 50 anti-PR3-ANCA positives, 32 males, 18 females, median age 63 years, range 17 to 83 years) and sera from 100 age- and sex-matched healthy volunteers were used as a 'training set' for the development of a ANCA pattern recognition algorithm module for the automated AKLIDES™ system. Sera were tested for MPO or PR3 ANCA by ELISA and line immunodot assay (LIA) (GA Generic Assays GmbH, Dahlewitz/Berlin, Germany).

As the 'test set', 342 serum samples from patients with AAV, other systemic rheumatic and infectious diseases as controls and from healthy individuals were used (Table 1). Patients were diagnosed based on typical disease history, characteristic clinical findings, and confirmed clinical histology according to the criteria of the 1992 Chapel Hill Consensus Conference, the consensus statement of 1999 and the 1990 American College of Rheumatology [2,4,18]. Serum samples were obtained from patients with a confirmed clinical diagnosis of GPA, EGPA or MPA irrespective of serology (that is, presence of ANCA was not used as a diagnostic criterion). All serum samples were taken at the time of diagnosis. Serum samples from patients with systemic lupus erythematosus (SLE) and rheumatoid arthritis (RA) were also used as disease controls. Disease extent was assessed using the Disease Extent Index (DEI) and disease activity using the Birmingham Vasculitis Activity Score (BVAS) at the time point when the sera were collected from the GPA, EGPA and MPA patients [19,20]. In total, 51 sera from patients with infectious disease (*Treponema pallidum *(*n *= 1), cytomegalovirus (*n *= 25), rubella virus (*n *= 5), *Toxoplasma gondii *(*n *= 16), hepatitis C (*n *= 1), Eppstein-Barr virus (*n *= 3) were included as disease controls in the test set. Furthermore, sera from 44 age- and sex-matched healthy volunteers were tested as control samples in the test set.

**Table 1:** Patient characteristics of the 'test set' comprising 342 serum samples.

Diagnosis	n (%)	age	gender f/m
GPA	59 (17.3)	21 - 81	36/23
EGPA	40 (11.7)	21 - 70	20/20
MPA	20 (5.8)	17 - 80	13/7
SLE	40 (11.7)	20 - 72	31/9
RA	30 (8.8)	40 - 81	23/7
RA and rheumatoid vasculitis	10 (2.9)	44 - 67	5/5
Cryoglobulinaemic vasculitis	10 (2.9)	43 - 78	7/3
Systemic sclerosis	38 (11.1)	35 - 84	27/11
Infectious diseases	51 (14.9)	5 - 86	46/5
Healthy controls	44 (12.9)	23 - 71	28/16

Patients with GPA consist of cases with generalized and localized GPA demonstrating inactive as well as active disease. Patients with EGPA cover cases with active and inactive disease. EGPA, eosinophilic granulomatosis with polyangiitis (Churg-Strauss Syndrome); GPA, granulomatosis with polyangiitis (Wegener's); f, female; m, male; MPA, microscopic polyangiitis; RA, rheumatoid arthritis; SLE, systemic lupus erythematosus.

The study received approval from the ethical committee of the Technical University of Dresden (EK226112006) and fulfilled the ethical guidelines of the most recent declaration of Helsinki. Written informed consent was obtained from each patient.

### Indirect immunofluorescence (IIF)

Employing IIF, ANCA were detected by running patient samples on ethN and formN according to the recommendations of the manufacturer (GA Generic Assays GmbH, Dahlewitz/Berlin, Germany). Briefly, fixed human neutrophils were incubated in a moist chamber at room temperature (RT) for 30 minutes with 25 μl of serum diluted 1:20. After washing, immune complexes were detected by incubating the samples with fluorescein isothiocyanate (FITC)-conjugated goat anti-human IgG for 30 minutes at RT. Samples were subsequently washed, embedded with a mounting medium containing 4',6-diamidino-2-phenylindol (DAPI) for nuclear staining, and analysed automatically by the novel ANCA pattern recognition algorithm module of AKLIDES™ (Medipan GmbH, Dahlewitz/Berlin, Germany) followed by manual interpretation with a routine fluorescence microscope (Carl Zeiss AG, Jena, Germany). To minimize subjectivity, manual reading was carried out always by two investigators (IK, TBr, TB, KG and EC).

### AKLIDES™ - technical system and pattern recognition algorithm software

The concept of the fully automated interpretation system AKLIDES™ for evaluation of ANCA IIF patterns is based on novel mathematical software algorithms for pattern recognition [12,21]. Neutrophils were assessed automatically using a motorized inverse microscope (IX81, Olympus Corporation, Tokyo, Japan) with a motorized scanning stage (IM120, Märzhäuser, Wetzlar, Germany); 400 nm and 490 nm light-emitting diodes (LED) (PrecisExcite, CoolLED, Andover, UK), and a charge-coupled device grey-scale camera (DX4, Kappa, Gleichen, Germany). The interpretation system is controlled by the AKLIDES™ software consisting of modules for device and autofocus control, image analysis, and pattern recognition algorithms. The novel autofocus based on Haralick's image characterization of objects through grey-scale transition using DAPI as fluorescent dye for focusing, quality evaluation, and object recognition.

Two-dimensional images were acquired using an objective with 40-fold magnification (Olympus semi-apochromat LUCPLFLN 40X, 0.60 NA, W.D. 2.7-4.0 mm). Fluorescence detection was performed using LED excitation with appropriate multiband filter for the DAPI and FITC dyes (DA/FI-A, Semrock, Rochester, USA). Single DAPI and FITC image were serially captured and stored in lossless compressed Tagged Image File (TIF) format.

**Figure 1:**
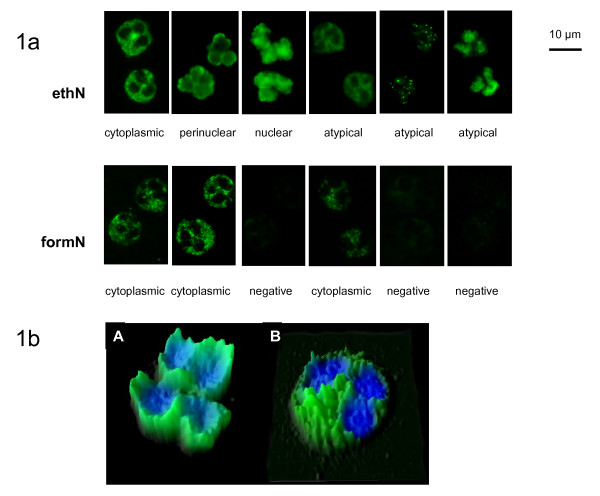
**ANCA patterns and recognition thereof by mathematical algorithms**. **(a) **Images (detail) of serum samples demonstrating C-ANCA, P-ANCA, and atypical ANCA patterns on ethN and formN taken automatically by AKLIDES™. Chromatin is stained by DAPI (blue) and specific ANCA interactions are revealed by FITC (green)-labeled secondary anti-human IgG. **(b) **Pattern recognition image of perinuclear (A) and cytoplasmic (B) specific staining of neutrophils used by the novel algorithms for pattern differentiation: DAPI (blue) and FITC fluorescence intensity signals (green) of respective images were combined and illustrated in three dimensions (x - object length, y - object width, light intensity of fluorescence signal). C-ANCA, cytoplasmic antineutrophil cytoplasmic antibody; ethN, ethanol-fixed neutrophils; formN, formalin-fixed neutrophils; P-ANCA, perinuclear antineutrophil cytoplasmic antibody.

To eliminate artifacts, an additional qualitative image analysis was performed by dividing the image content into tiles of equal size with subsequent calculation of tile sharpness and homogeneity. Object segmentation was conducted by histogram-based threshold algorithm followed by watershed transformation [21]. Cell aggregates were excluded by analyzing convexity of objects. Segmented granulocytes were characterized by regional, topological, and texture/surface descriptors by employing DAPI and FITC image data (Figure 1a). A minimum of 20 granulocytes were counted at each slide.

### Statistical analysis

Fisher's exact test was used to check the differences between the two classification systems. To test for the strength of agreement, inter-rater agreement statistics was conducted. McNemar test was performed to check the difference for paired proportions. *P *values of less than 0.05 were considered as or to be significant. Calculations were performed by using MedCalc™ statistical software (MedCalc, Mariakerke, Belgium).

## Results

### Development of ANCA pattern recognition algorithms on AKLIDES™

For the automated interpretation of ANCA IIF patterns and evaluation of its diagnostic performance, a novel ANCA pattern recognition algorithm software module was established for the multicolor-fluorescence reading system AKLIDES™ employing topographical, texture, boundary, and regional descriptors for image analysis. Both ANCA pattern detection and description follow a two-step approach. At first, DAPI staining was used for image focusing, checking of scene quality, and neutrophil identification. Afterwards, signal intensity and pattern classification for appropriate neutrophils were calculated for FITC fluorescence (Figure 1a, b).

First, a 'training set' of 70 ANCA positive samples with distinct ANCA specificities (anti-MPO-ANCA, *n *= 20 and anti-PR3-ANCA, *n *= 50) and 100 healthy controls was used for the development and refinement of pattern recognition algorithms for the new ANCA pattern recognition algorithm module. The analysis of IIF patterns by image-processing algorithms was separated into two profiles for the recognition of ANCA IIF patterns on ethN and formN. Furthermore, a differentiation between positive and negative samples (quantitative threshold values are given in Table 2) and additionally between the two different basic ANCA IIF patterns cytoplasmic and nuclear/perinuclear (Figure 1a, b) was implemented. Five images per sample were taken automatically. The images were focused employing the DAPI fluorescence and the pattern recognition was performed by adding FITC fluorescence signals of at least 20 neutrophils. Running the 'training set' samples, a 100% consistency was achieved regarding positive/negative discrimination and determination of ANCA IIF patterns by using the novel pattern recognition algorithms (data not shown) in comparison to manual interpretation.

**Table 2:** Comparison of manual and automated positive/negative discrimination assessing fluorescence intensity of 342 samples on ethN and formN patterns.

	**ethN**	**Automated interpretation by AKLIDES™**					
		
	**n (%)**	**-**	**+/-**	**+**	**++**	**+++**	**++++**
	
	-	133 (38.9)	1 (0.3)	0 (0)	0 (0)	0 (0)	0 (0)
	+/-	24 (7.0)	29 (8.5)	12 (3.5)	0 (0)	0 (0)	0 (0)
	+	0 (0)	4 (1.2)	44 (12.9)	11 (3.2)	0 (0)	0 (0)
	++	0 (0)	0 (0)	4 (1.2)	34 (9.9)	9 (2.6)	0 (0)
	+++	0 (0)	0 (0)	0 (0)	3 (0.9)	16 (4.6)	5 (1.5)
	++++	0 (0)	0 (0)	0 (0)	0 (0)	0 (0)	13 (3.8)
Visual interpretation	formN						
		
	n (%)	-	+/-	+	++	+++	++++
	
	-	225 (65.8)	8 (2.3)	2 (0.6)	2 (0.6)	0 (0)	0 (0)
	+/-	9 (2.6)	22 (6.4)	5 (1.5)	0 (0)	0 (0)	0 (0)
	+	0 (0)	5 (1.5)	25 (7.3)	6 (1.7)	0 (0)	0 (0)
	++	0 (0)	0 (0)	2 (0.6)	20 (5.8)	1 (0.3)	0 (0)
	+++	0 (0)	0 (0)	0 (0)	1 (0.3)	6 (1.7)	0 (0)
	++++	0 (0)	0 (0)	0 (0)	0 (0)	0 (0)	3 (0.9)

ANCA IIF patterns were first interpreted automatically with AKLIDES™ followed by manual reading with a routine fluorescence microscope. Both interpretation methods demonstrated very good agreement on ethN and formN (κ = 0.955, 95% confidence interval (CI): 0.944 to 0.965; κ = 0.929, 95% CI: 0.895 to 0.964; respectively). Quantitative fluorescence intensity threshold values for ethN: < 15 negative (-); ≥ 15 borderline (+/-) < 40; ≥ 40 weak positive (+) < 150; ≥ 150 positive (++) < 400; ≥ 400 strong positive (+++) < 700; ≥ 700 very strong positive (++++). Quantitative fluorescence intensity threshold values for formN: < 20 negative (-); ≥ 20 borderline (+/-) < 30; ≥ 30 weak positive (+) < 100; ≥ 100 positive (++) < 300; ≥ 300 strong positive (+++) < 600; ≥ 600 very strong positive (++++). ANCA, antineutrophil cytoplasmic antibody; ethN, ethanol-fixed neutrophils; formN, formalin-fixed neutrophils; IIF, indirect immunofluorescence.

### Signal variability of automated ANCA pattern interpretation

In contrast to visual reading, measurement of quantitative fluorescence signals for ANCA IIF pattern interpretation provides for the first time the basis for objective assay performance assessment by determination of assay variability. Inter-assay coefficients of variation (CV) were determined by running six ANCA positive samples with differing ANCA titers in three different runs. The functional assay sensitivity representing the lowest ANCA concentration with a CV of lower than 20% was determined at a fluorescence intensity level of 40 AU for ethN and 30 AU for formN (data not shown)

### Positive/negative discrimination of ANCA by automated interpretation

**Figure 2:**
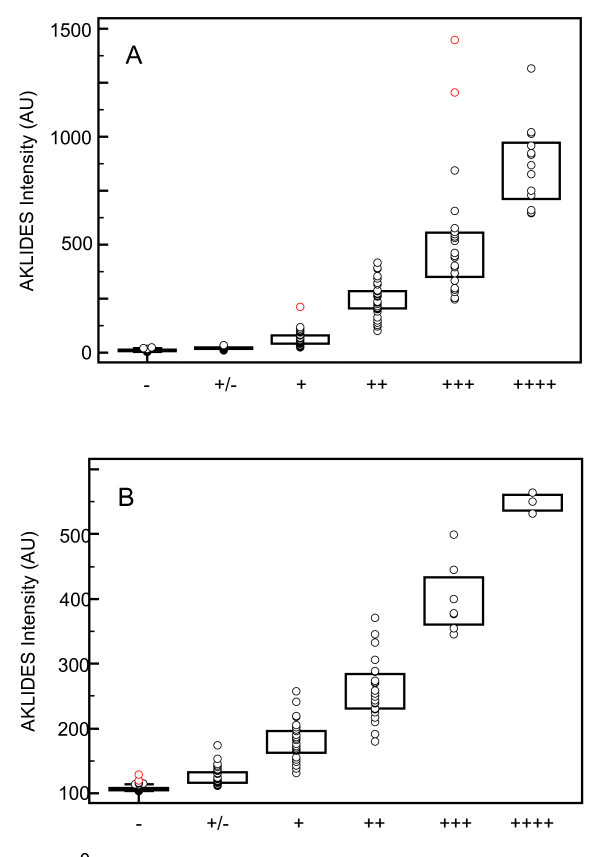
**Fluorescence intensity (AU) obtained by automated reading of AKLIDES™ compared to visual interpretation of ANCA IIF pattern images on ethN (A) and formN (B) investigating serum samples of the 'test set'**. ANCA, antineutrophil cytoplasmic antibody; ethN, ethanol-fixed neutrophils; formN, formalin-fixed neutrophils; IIF, indirect immunofluorescence.

The established ANCA IIF pattern recognition algorithm module was evaluated with the 'test set' comprising 342 patient and control sera (Table 1) to assess the ability to differentiate between positive and negative samples. To compare manual and automated positive/negative discrimination, ANCA IIF patterns were interpreted automatically with AKLIDES™ followed by visual interpretation with a routine fluorescence microscope (Figure 1a). Appropriate ranges of quantitative fluorescence intensity data obtained with the 'training set' samples with AKLIDES™ were used to classify automated findings into negative (-), borderline (+/-), weak positive (+), positive (++), strong positive (+++), and very strong positive (++++) (Table 2). The threshold values were used for the positive/negative classification of the samples in the test set (Figure 2, Table 2).

Counting borderline samples as negative, the differences in positive and negative findings of 2.34% (95% confidence interval (CI): -0.22 to 4.0%) on ethN and 0.58% (95% CI: -1.57 to 2.44%) on formN between both interpretation methods were not significant according to McNemar's test (*P *= 0.0768, 0.7744, respectively). Applying inter-rater agreement statistics for testing the strength of agreement between two classifications, Cohen's kappa (κ) values above 0.8 on ethN and formN were obtained (κ = 0.955, 95% CI: 0.944 to 0.965; κ = 0.929, 95% CI: 0.895 to 0.964; respectively). Thus, visual and automated interpretation revealed a very good strength of agreement for the 'test set' sera.

### ANCA IIF pattern recognition by automated interpretation

Employing the novel pattern recognition algorithms, 'test set' patient samples (*n *= 342) were interpreted automatically with AKLIDES™ followed by visual interpretation to compare the ANCA IIF pattern findings. Four different patterns such as cytoplasmic, nuclear/perinuclear, atypical, and negative were used to classify automated and visual findings on ethN for inter-rater agreement analysis (Table 3). Automated and visual interpretation of ANCA pattern demonstrated a good agreement with a κ value of 0.746 on ethN (95% CI: 0.667 to 0.825). The main differences between visual and automated interpretation for IIF patterns on ethN were found for visual findings regarding cytoplasmic and atypical patterns. Out of 88 cytoplasmic pattern findings by visual interpretation, the automated reading defined 22 (25.0%) as negative results. All these 22 IIF images demonstrated a borderline cytoplasmic immunofluorescence on AKLIDES™ that did not reach the threshold for positivity used by the pattern recognition algorithms. Nevertheless, there was no significant difference in the number of cytoplasmic patterns like for all other patterns determined on ethN regarding automated and visual interpretation (87/342 by visual vs. 62/342 by automated interpretation, *P *= 1.00). Moreover, out of 55 atypical patterns determined visually, 10 (18.2%) were defined as nuclear pattern using the automated pattern recognition algorithms.

**Table 3:** Comparison of visual and automated interpretation of IIF ANCA patterns of the 'test set' patients (*n *= 342) on ethN and formN.

	**ethN**	**Automated interpretation by AKLIDES™**			
		
	**n (%)**	**cytoplasmic**	**nuclear**	**atypical**	**negative**
	
	cytoplasmic	59 (17.2)	0 (0)	7 (2.0)	22 (6.4)
	nuclear	0 (0)	61 (17.8)	4 (1.2)	0 (0)
	atypical	3 (0.9)	10 (2.9)	40 (11.7)	2 (0.6)
	negative	0 (0)	0 (0)	1 (0.3)	133 (38.9)
	
Visual interpretation	formN				
	n (%)	cytoplasmic	atypical	negative	
			
	cytoplasmic	91 (26.6)	0 (0)	9 (2.6)	
	atypical	8 (2.3)	0 (0)	0 (0)	
	negative	9 (2.6)	0 (0)	225 (65.8)	

ANCA IIF patterns were first read automatically with AKLIDES™ followed by manual interpretation with a routine fluorescence microscope. Both interpretation methods demonstrated good agreement on ethN and formN (κ = 0.746, 95% CI: 0.667 to 0.825; κ = 0.847, 95% CI: 0.79 to 0,904; respectively). ANCA, antineutrophil cytoplasmic antibody; ethN, ethanol-fixed neutrophils; formN, formalin-fixed neutrophils; IIF, indirect immunofluorescence.

Due to the impaired mobility of MPO during formalin fixation of neutrophils, only three kinds of patterns were classified on formN (cytoplasmic, atypical, and negative). Comparison of automated and visual interpretation on formN revealed a very good agreement (κ = 0.847, 95% CI: 0.790 to 0,904). Out of 100 cytoplasmic pattern findings by visual interpretation, the automated reading defined nine (9.0%) as negative results. These nine IIF images demonstrated also a borderline cytoplasmic immunofluorescence that did not reach the threshold for positivity used by the pattern recognition algorithms. In contrast, out of 234 negative samples by visual interpretation, nine (3.8%) IIF pattern images were interpreted as cytoplasmic pattern. All eight atypical patterns by visual interpretation were classified as cytoplasmic pattern by automated interpretation, which was the only significant difference between both methods for pattern recognition on formN (*P *= 0.0075).

### ANCA IIF pattern recognition comparison in different clinical entities

**Figure 3:**
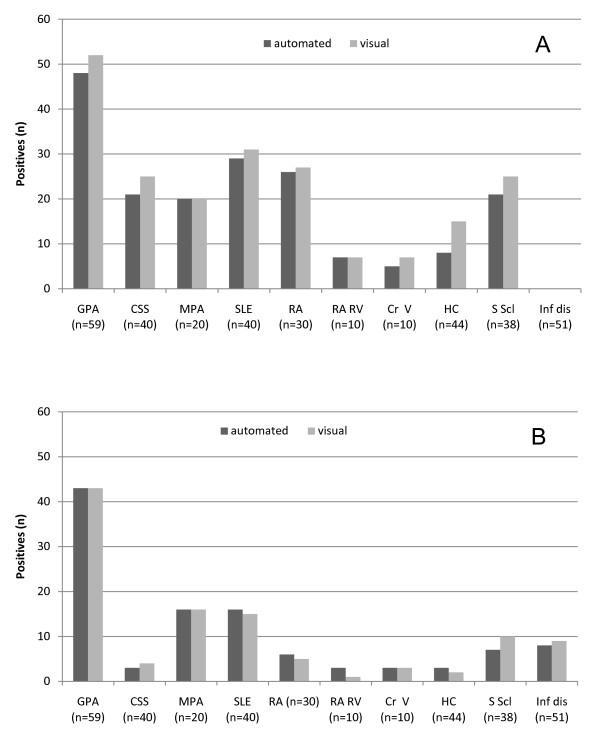
**Comparison of automated and visual interpretation of ANCA IIF patterns in patient and control samples of the 'test set' on ethN (A) and formN (B)**. Patterns of ethN and formN were first detected automatically by AKLIDES™ and then interpreted manually by a routine fluorescence microscope. GPA, granulomatosis with polyangiitis (Wegener's) (*n *= 59); EGPA, eosinophilic granulomatosis with polyangiitis (Churg-Strauss Syndrome) (*n *= 40); MPA, microscopic polyangiitis (*n *= 20); SLE, systemic lupus erythematosus (*n *= 40); RA, rheumatoid arthritis (*n *= 30); RA RV, rheumatoid arthritis with rheumatoid vasculitis (*n *= 10); Cr V, cryoglobulinaemic vasculitis (*n *= 10); HC, healthy control (*n *= 44); S Scl, systemic sclerosis (*n *= 38), Inf dis, infectious disease (*n *= 51).

Automated and visual interpretation of ANCA IIF patterns on ethN and formN included different patient and control group samples (Figure 3). Investigating sera from patients with GPA (generalized and localized with active and inactive disease state), EGPA (active and inactive), MPA, SLE, RA, rheumatoid vasculitis, cryoglobulinaemic vasculitis, systemic sclerosis, infectious disease and healthy controls, no significant difference between both interpretation methods was established for the respective clinical entities and controls (*P *> 0.05, respectively).

## Discussion

Testing for ANCA plays a pivotal role in the serological diagnosis of AAV [22]. In accordance with international guidelines, IIF and independent detection techniques like ELISA should be used for the assessment of ANCA [4,23]. Interestingly, IIF has been reported to be used as a screening method due to its high sensitivity followed by anti-PR3-ANCA and anti-MPO-ANCA ELISAs as confirmatory tests under routine laboratory conditions due to its high sensitivity and negative predictive value [24]. In contrast to the ELISA technique, IIF is prone to human bias and ANCA IIF image interpretation has been not automated until recently [16]. Thus, the recommended combination of IIF and confirmatory testing requires a high level of expert knowledge and lacks standardization [25]. Several studies tried to demonstrate that ELISA technology is better adaptable for standardization of ANCA assessment and has a lower lab-to-lab variability compared to IIF [26-28]. However, most commercially available ELISAs have been reported to be inferior to IIF in terms of sensitivity [29]. Therefore, automated interpretation of ANCA IIF patterns as demonstrated for ANA detection by novel pattern recognition algorithms could provide a cost-efficient and standardized alternative approach for ANCA screening [13-16]. In particular, regarding routine laboratories with large numbers of ANCA determinations, automated interpretation would overcome shortcomings of IIF such as high level of manual work and exceeding data management [13,16].

Several studies have confirmed the usefulness of automated IIF systems for objective ANA pattern interpretations recently, providing the basis for the employment of IIF as gold standard for ANA testing as required by the American College of Rheumatology even under differing routine conditions [13,14,16,30].

In contrast to ANA detection on HEp-2 cells, polymorphonuclear granulocytes are characterized by varying shapes of the nucleus, which is usually lobed into three segments. Therefore, algorithms for identification of granulocyte staining patterns proved to be more complex compared to those for HEp-2 cells. Furthermore, ethN and formN show different boundary characteristics with DAPI staining. Consequently, the type of fixation needs to be taken into account assessing signal intensity and pattern classification to adapt the algorithms to the changed morphology.

Due to polymorphic nucleus and cytoplasmic ANCA staining patterns, signal assessment and pattern classification cannot be performed simply inside or outside of the DAPI positive area as described for ANA pattern detection [12,21]. Cytoplasmic and perinuclear ANCA patterns need to be detected and identified in border regions of the nucleus, rendering detection of ANCA more complex and challenging for automation.

As a fact, our study did not establish a significant difference between automated and visual ANCA IIF detection investigating patients with AAV and disease controls. Furthermore, the positive/negative discrimination and ANCA pattern assessment demonstrated a very good agreement for ethN and formN, confirming the usefulness of pattern recognition algorithms for the automated interpretation of IIF patterns. In contrast to the work of Melegari *et al. *reporting a very good consistency for positive/negative discrimination by automated and visual interpretation in a review, in this study ANCA patterns were also analyzed on neutrophils with two different fixation methods.

Moreover, this is the first study on ANCA IIF pattern reading, reporting quantitative data for interassay variability. As shown for anti-dsDNA and ANA detection by IIF recently, this approach allows determining a functional assay sensitivity giving the lowest fluorescence intensity with an interassay variability of equal or less than 20% [15,31,32]. This creates the opportunity for a standardized cutoff determination improving the test accuracy for borderline samples and might provide a better reporting of ANCA IIF results to clinicians in order to support the progress in the treatment of AAV [33]. Relying on quantitative data for ANCA IIF reading may even give the opportunity to report findings for different test-result intervals as proposed recently for ANCA testing by ELISA [34].

## Conclusions

Novel ANCA pattern recognition algorithms are a very useful tool for the automated reading of ANCA patterns on ethN and formN and can further improve the standardization efforts for the detection of ANCA. The automated evaluation is prone to render the comparison of diagnostic data possible. This is most important for clinical studies but also for diagnostic purposes and in clinical research. Automated pattern interpretation demonstrated high diagnostic performance for the assessment of ANCA and revealed no difference to visual reading. Further studies are warranted to evaluate this novel fully automated approach for automated software-based screening of ANCA in patients with suspected AAV.

## Abbreviations

ANA: antinuclear antibody; ANCA: antineutrophil cytoplasmic antibody; AAV: ANCA-associated vasculitides; C-ANCA: cytoplasmic antineutrophil cytoplasmic antibody; CI: confidence interval; EGPA: eosinophilic granulomatosis with polyangiitis (Churg-Strauss Syndrome); ELISA: enzyme-linked immunosorbent assay; ethN: ethanol-fixed neutrophils; formN: formalin-fixed neutrophils; GPA: granulomatosis with polyangiitis (Wegener's); IIF: indirect immunofluorescence; κ: Cohen's kappa; MPA: microscopic polyangiitis; MPO: myeloperoxidase; P-ANCA: perinuclear antineutrophil cytoplasmic antibody; PR3: proteinase 3; RA: rheumatoid arthritis; SLE: systemic lupus erythematosus.

## Competing interests

Dirk Roggenbuck has a management role and is a shareholder of GA Generic Assays GmbH and Medipan GmbH. Both companies are diagnostic manufacturers. All other authors declare that they have no competing financial interests.

## Authors' contributions

IK, TBr, TB, KG, and EC carried out indirect immunofluorescence assays. RH developed the pattern recognition algorithms. MC, FP, and MOB provided the serum samples from patients with infectious diseases and carried out the respective laboratory investigations. KC, DRe, UA, EC, and DRo conceived of the study, and participated in its design and coordination and helped to draft the manuscript. All authors read and approved the final manuscript.

## References

[B1] Bosch X Guilabert A Font J Antineutrophil cytoplasmic antibodies Lancet 2006 14 404 18 10.1016/S0140-6736(06)69114-9 16876669

[B2] Jennette JC Falk RJ Andrassy K Bacon PA Churg J Gross WL Hagen EC Hoffman GS Hunder GG Kallenberg CG Nomenclature of systemic vasculitides. Proposal of an international consensus conference Arthritis Rheum 1994 14 187 92 10.1002/art.1780370206 8129773

[B3] Falk RJ Gross WL Guillevin L Hoffman GS Jayne DR Jennette JC Kallenberg CG Luqmani R Mahr AD Matteson EL Merkel PA Specks U Watts RA Granulomatosis with polyangiitis (Wegener's). an alternative name for Wegener's granulomatosis Arthritis Rheum 2011 14 863 4 10.1002/art.30286 21374588

[B4] Savige JF Gillis DF Benson E Davies DF Esnault VF Falk RJ Hagen EC Jayne D Jennette JC Paspaliaris B Pollock W Pusey C Savage CO Silvestrini R van der Woude F Wieslander J Wiik A International Consensus Statement on Testing and Reporting of Antineutrophil Cytoplasmic Antibodies (ANCA) Am J Clin Pathol 1999 14 507 13 10191771 10.1093/ajcp/111.4.507

[B5] Wiik A Autoantibodies in vasculitis Arthritis Res Ther 2003 14 147 52 12723981 10.1186/ar758 PMC165052

[B6] van der Woude FJ Rasmussen N Lobatto S Wiik A Permin H van Es LA van der Giessen M van der Hem GK The TH Autoantibodies against neutrophils and monocytes: tool for diagnosis and marker of disease activity in Wegener's granulomatosis Lancet 1985 14 425 9 2857806 10.1016/s0140-6736(85)91147-x

[B7] Conrad K Schmechta H Klafki A Lobeck G Uhlig HH Gerdi S Henker J Serological differentiation of inflammatory bowel diseases Eur J Gastroenterol Hepatol 2002 14 129 35 10.1097/00042737-200202000-00006 11981336

[B8] Joossens S Daperno M Shums Z Van SK Goeken JA Trapani C Norman GL Godefridis G Claessens G Pera A Pierik M Vermeire S Rutgeerts P Bossuyt X Interassay and interobserver variability in the detection of anti-neutrophil cytoplasmic antibodies in patients with ulcerative colitis Clin Chem 2004 14 1422 5 10.1373/clinchem.2004.032318 15277351

[B9] Terjung B Worman HJ Herzog V Sauerbruch T Spengler U Differentiation of antineutrophil nuclear antibodies in inflammatory bowel and autoimmune liver diseases from antineutrophil cytoplasmic antibodies (p-ANCA) using immunofluorescence microscopy Clin Exp Immunol 2001 14 37 46 10.1046/j.1365-2249.2001.01649.x 11678897 PMC1906166

[B10] Craig WY Ledue TB Collins MF Meggison WE Leavitt LF Ritchie RF Serologic associations of anti-cytoplasmic antibodies identified during anti-nuclear antibody testing Clin Chem Lab Med 2006 14 1283 6 17032143 10.1515/CCLM.2006.232

[B11] McLaren JS Stimson RH McRorie ER Coia JE Luqmani RA The diagnostic value of anti-neutrophil cytoplasmic antibody testing in a routine clinical setting QJM 2001 14 615 21 10.1093/qjmed/94.11.615 11704691

[B12] Hiemann R Buttner T Krieger T Roggenbuck D Sack U Conrad K Challenges of automated screening and differentiation of non-organ specific autoantibodies on HEp-2 cells Autoimmun Rev 2009 14 17 22 10.1016/j.autrev.2009.02.033 19245860

[B13] Egerer K Roggenbuck D Hiemann R Weyer MG Buttner T Radau B Krause R Lehmann B Feist E Burmester GR Automated evaluation of autoantibodies on human epithelial-2 cells as an approach to standardize cell-based immunofluorescence tests Arthritis Res Ther 2010 14 R40 10.1186/ar2949 20214808 PMC2888187

[B14] Kivity S Gilburd B Agmon-Levin N Carrasco MG Tzafrir Y Sofer Y Mandel M Buttner T Roggenbuck D Matucci-Cerinic M Danko K Hoyos ML Shoenfeld Y A novel automated indirect immunofluorescence autoantibody evaluation Clin Rheumatol 2011 14 503 9 22057233 10.1007/s10067-011-1884-1

[B15] Roggenbuck D Reinhold D Hiemann R Anderer U Conrad K Standardized detection of anti-ds DNA antibodies by indirect immunofluorescence - a new age for confirmatory tests in SLE diagnostics Clin Chim Acta 2011 14 2011 2 10.1016/j.cca.2011.07.005 21782805

[B16] Melegari A Bonaguri C Russo A Luisita B Trenti T Lippi G A comparative study on the reliability of an automated system for the evaluation of cell-based indirect immunofluorescence Autoimmun Rev 2012 14 713 16 10.1016/j.autrev.2011.12.010 22269861

[B17] Boomsma MM Damoiseaux JG Stegeman CA Kallenberg CG Patnaik M Peter JB Tervaert JW Image analysis: a novel approach for the quantification of antineutrophil cytoplasmic antibody levels in patients with Wegener's granulomatosis J Immunol Methods 2003 14 27 35 10.1016/S0022-1759(02)00273-9 12609530

[B18] Leavitt RY Fauci AS Bloch DA Michel BA Hunder GG Arend WP Calabrese LH Fries JF Lie JT Lightfoot RW Jr The American College of Rheumatology 1990 criteria for the classification of Wegener's granulomatosis Arthritis Rheum 1990 14 1101 7 2202308 10.1002/art.1780330807

[B19] deGroot K Gross WL Herlyn K Reinhold-Keller E Development and validation of a disease extent index for Wegener's granulomatosis Clin Nephrol 2001 14 31 8 11200865

[B20] Luqmani RA Bacon PA Moots RJ Janssen BA Pall A Emery P Savage C Adu D Birmingham Vasculitis Activity Score (BVAS) in systemic necrotizing vasculitis QJM 1994 14 671 8 7820541

[B21] Hiemann R Hilger N Sack U Weigert M Objective quality evaluation of fluorescence images to optimize automatic image acquisition Cytometry A 2006 14 182 4 16496376 10.1002/cyto.a.20224

[B22] Bosch X Font J Mirapeix E Revert L Urbano-Marquez A Ingelmo M Anti-neutrophil cytoplasmic autoantibodies (ANCA): antigenic specificities and clinical associations Adv Exp Med Biol 1993 14 281 6 8296618 10.1007/978-1-4757-9182-2_42

[B23] Savige J Gillis D Benson E Davies D Esnault V Falk RJ Hagen EC Jayne D Jennette JC Paspaliaris B Pollock W Pusey C Savage CO Silvestrini R van der Woude F Wieslander J Wiik A International Consensus Statement on Testing and Reporting of Antineutrophil Cytoplasmic Antibodies (ANCA) Am J Clin Pathol 1999 14 507 13 10191771 10.1093/ajcp/111.4.507

[B24] Tsiveriotis K Tsirogianni A Pipi E Soufleros K Papasteriades C Antineutrophil cytoplasmic antibodies testing in a large cohort of unselected greek patients Autoimmune Dis 2011 14 626495 21687647 10.4061/2011/626495 PMC3112505

[B25] Csernok E Holle JU Twenty-eight years with antineutrophil cytoplasmic antibodies (ANCA): how to test for ANCA - evidence-based immunology? Springer-Verlag 2010 14 39 43 10.1007/s13317-010-0007-3 PMC4389065 26000106

[B26] Roggenbuck D Buettner T Hoffmann L Schmechta H Reinhold D Conrad K High-sensitivity detection of autoantibodies against proteinase-3 by a novel third-generation enzyme-linked immunosorbent assay Ann N Y Acad Sci 2009 14 41 6 10.1111/j.1749-6632.2009.04649.x 19758130

[B27] Hagen EC Andrassy K Csernok E Daha MR Gaskin G Gross WL Hansen B Heigl Z Hermans J Jayne D Kallenberg CG Lesavre P Lockwood CM Lüdemann J Mascart-Lemone F Mirapeix E Pusey CD Rasmussen N Sinico RA Tzioufas A Wieslander J Wiik A Van der Woude FJ Development and standardization of solid phase assays for the detection of anti-neutrophil cytoplasmic antibodies (ANCA). A report on the second phase of an international cooperative study on the standardization of ANCA assays J Immunol Methods 1996 14 1 15 10.1016/0022-1759(96)00111-1 8841439

[B28] Csernok E Holle J Hellmich B Willem J Tervaert C Kallenberg CG Limburg PC Niles J Pan G Specks U Westman K Wieslander J De Groot K Gross WL Evaluation of capture ELISA for detection of antineutrophil cytoplasmic antibodies directed against proteinase 3 in Wegener's granulomatosis: first results from a multicentre study Rheumatology (Oxford) 2004 14 174 80 14585921 10.1093/rheumatology/keh028

[B29] Csernok E Ahlquist D Ullrich S Gross WL A critical evaluation of commercial immunoassays for antineutrophil cytoplasmic antibodies directed against proteinase 3 and myeloperoxidase in Wegener's granulomatosis and microscopic polyangiitis Rheumatology (Oxford) 2002 14 1313 7 10.1093/rheumatology/41.11.1313 12422006

[B30] Meroni PL Schur PH ANA screening: an old test with new recommendations Ann Rheum Dis 2010 14 1420 2 10.1136/ard.2009.127100 20511607

[B31] Zöphel K Wunderlich G Kotzerke J von Landenberg P Roggenbuck D M22 based (manual) ELISA for TSH-receptor antibody (TRAb) measurement is more sensitive than 2nd generation TRAb assays Clin Chim Acta 2009 14 266 10.1016/j.cca.2009.01.022 19361467

[B32] Willitzki A Hiemann R Peters V Sack U Schierack P Rödiger S Anderer U Conrad K Bogdanos DP Reinhold D Roggenbuck D New platform technology for comprehensive serological diagnostics of autoimmune diseases Clin Dev Immunol 2012 14 284740 23316252 10.1155/2012/284740 PMC3536031

[B33] Smith RM Jones RB Jayne DR Progress in treatment of ANCA-associated vasculitis Arthritis Res Ther 2012 14 210 10.1186/ar3797 22569190 PMC3446448

[B34] Vermeersch P Blockmans D Bossuyt X Use of likelihood ratios can improve the clinical usefulness of enzyme immunoassays for the diagnosis of small-vessel vasculitis Clin Chem 2009 14 1886 8 10.1373/clinchem.2009.130583 19643836

